# The presence of cariogenic and periodontal pathogens in the oral cavity of one-year-old infants delivered pre-term with very low birthweights: a case control study

**DOI:** 10.1186/1472-6831-14-109

**Published:** 2014-09-01

**Authors:** Vlasta Merglova, Romana Koberova-Ivancakova, Zdenek Broukal, Jiri Dort

**Affiliations:** 1 Department of Dentistry, Faculty of Medicine in Pilsen, Charles University in Prague, Alej Svobody 80, 304 60 Pilsen, Czech Republic; 2 Department of Dentistry, Faculty of Medicine in Hradec Kralove, Charles University in Prague, Sokolská 581, 500 05 Hradec Kralove, Czech Republic; 3 Institute of Clinical and Experimental Dental Medicine of Charles University in Prague, Karlovo nam 554/32, 120 00 Prague, Czech Republic; 4 Department of Neonatology, Faculty Hospital in Pilsen, Alej Svobody 80, 304 60 Pilsen, Czech Republic

**Keywords:** Cariogenic microbes, Dental caries, Periodontal pathogens, Pre-term infants, S. mutans

## Abstract

**Background:**

Recently, the dental literature has focused mainly on the microbial colonization of healthy full-term infants and their mothers or caretakers. However, oral microbial acquisition by premature infants has not been adequately investigated, and the correlation between pre-term birth and the presence of cariogenic and periodontal pathogens has not been determined. The aim of this study was to identify the presence and quantities of representative cariogenic and periodontal pathogens in the oral cavities of 12-month-old infants and compare the occurrence of these microbes between a cohort of pre-term infants with very low birthweights and a control cohort comprising full-term infants.

**Methods:**

The research cohort was composed of 69 one-year-old infants, of whom 24 were born prematurely with very low birthweights and 45 of whom were carried to full term. Information regarding the infants’ gestational age, mode of delivery, general health status, birthweight and antibiotic use were obtained from hospital records and through oral interviews. At 12 months of age, both groups of infants were examined, and unstimulated saliva samples from the dorsum of the tongue and dental plaque samples were collected. The microorganisms (*Streptococcus mutans, Lactobacillus spp., Actinomyces spp., Aggregatibacter actinomycetemcomitans, Porphyromonas gingivalis, Tannerella forsythia, Treponema denticola, Peptostreptococcus micros, Prevotella intermedia, Fusobacterium nucleatum*) were identified and their quantities were evaluated using a PCR-based method. The chi-squared and Fisher’s factorial tests were used for the statistical evaluations.

**Results:**

The infants had a high prevalence of cariogenic microbes and of *Fusosbacterium nucleatum* and *Aggregatibacter actinomycetemcomitans*. Cariogenic microbes were detected in 91.7% of the very low birthweight infants and in all full-term infants. Periodontal pathogens were present in 83% of the pre-term infants and in 96% of the full-term infants. A significant difference was found between the cohorts in terms of the presence of *S. mutans*. Most of the very low birthweight infants had negative values of this microbe, while the full-term infants had positive values.

**Conclusions:**

This study confirms the early transmission of representative cariogenic and periodontal pathogens to the oral cavity of one-year-old infants and a higher prevalence of *S. mutans* in full-term infants than in premature infants.

## Background

Pre-term birth and low birthweight are major causes of mortality and morbidity in neonates across the world. According to the definition of the World Health Organization [1], a pre-term infant is defined as being born before gestational week 37 or having low birthweight. A low birthweight is defined as weighting less than 2500 g, regardless of the gestational age. Low birthweight neonates are further subdivided into very low birthweight infants (VLBW), with birthweights < 1500 g, and extremely low birthweight infants, with birthweights < 1000 g. Pre-term delivery accounts for approximately 6% of all live births in developed Western European countries [2]. Premature low birthweight infants have a shorter prenatal period and are predisposed to various perinatal and neonatal complications and developmental problems that can affect their general growth and progress during infancy and throughout childhood. Low birthweight is closely related to infant perinatal mortality and to an increased risk of early and late morbidity.

Premature childbirth can be linked to the occurrence of periodontal diseases in pregnant women. It is believed that periodontal pathogens or the inflammatory mediators they induce reach the maternal reproductive system through the bloodstream and elicit an inflammatory cascade that gives rise to pre-term delivery [3]. According to some authors [4,5], certain oral Gram-negative bacteria create a cumulative effect that is sufficient to trigger early delivery, which represents the last step to produce a low birthweight labor. In the studies by Ercan et al. [5] and Gonzales-Marin et al. [6], the anaerobic microbe *F. nucleatum* was isolated from the amniotic fluid, placenta and chorioamnionic membranes of women delivering prematurely. Contrary to the work of Heimonen et al. [7], a causal linkage between a woman’s oral health status and her experience with pre-term or full-term births cannot be assumed.

Recently, the dental literature has focused on the microbial colonization of healthy full-term infants and their mothers or other caretakers. Authors [8-10] have emphasized microbial colonization and the transmission of cariogenic microbes in the oral cavity, as well as the role of *S. mutans* in the development of early childhood caries; however, oral microbial acquisition by premature infants with very low birthweights has not been adequately investigated.

We hypothesized that infants born pre-term would have different levels of oral cavity colonization with cariogenic and periodontal pathogens compared to full-term infants. We predicted that the earlier microbial colonization in the pre-term infants may be due to differences in their immune systems. The aim of this study was to determine the presence and values of the main known cariogenic and periodontal pathogens in the oral cavities of one-year-old infants without caries, and to compare the findings in pre-term very low birthweight infants with full-term infants.

## Methods

This case control study is a part of long-term research project supported by the Ministry of Health of the Czech Republic performed at the Department of Dentistry and Neonatology Faculty of Medicine in Pilsen, Charles University in Prague, Czech Republic. We recruited all one-year-old infants consecutively referred from the Department of Neonatology for examination during the study period between 1 January 2013 and 31 December 2013. Personal information including gestational age, mode of delivery, general health status, antibiotic use and medical history were obtained through oral interviews and from hospital records. The gestational age was estimated from the reported date of the mother’s last menstruation. The infants were divided into two groups: group A comprised infants delivered pre-term with very low birthweight, and group B comprised full-term infants. The presence of deciduous incisors in the oral cavity was the inclusion criterion for all infants, in addition to gestational age < 37 weeks and birthweight < 1500 g for group A and gestational age > 37 weeks and birthweight > 2500 g for group B. The inclusion criteria also included middle –class socioeconomic status for both groups. Infants in groups A and B meeting any of the following conditions were excluded from the study: 1) contemporary antibiotic treatment; 2) antibiotic treatment within 6 months prior to the clinical and microbiological examination; 3) systemic disease or immunological deficiency in full-term infants; and 4) any type of medication. Of the 87 examined one-year-old Caucasian infants, 69 were selected based on the inclusion and exclusion criteria. A definitive research cohort was composed of 69 one-year-old orally healthy Caucasian infants, 24 of whom (case group A) were born prematurely with very low birthweights and 45 of whom (control group B) were born at full term. The characteristics of the infant groups are reported in Table 1. The mean gestational age and birthweight of the infants in group A were 29.7 wks and 1154.9 g, respectively. Group A was characterized by polymorbidity (Table 2). Infants in group B were healthy individuals without a history of serious general disease with a mean gestational age of 39.8 wks and birthweight of 3254.7 g. During the neonatal period, all pre-term infants received antibiotic treatment consisting of intravenous ampicillin and gentamicin for 5 – 7 days.

**Table 1:** Characteristics of the research groups

**Group**	**Mean birthweight (g)**	**SD**	**Mean gestational age (weeks)**	**SD**	**Mode of delivery**	
					**Vaginal**	**Caesarean section**
Group A	1154.9	234.08	29.7	2.7	6 (25%)	18 (75%)
Group B	3254.7	392.7	39.8	1.0	33 (73.3%)	12 (26.7%)

**Table 2:** The main diseases of pre-term infants in neonatal period

**Disease**	**Number of affected preterm neonates**	**%**
Respiratory distress syndrome	23	95.8
Hyperbilirubinemia	18	75.0
Anemia	16	66.6
Patent ductus arteriosus	8	33.3
Adnate infection	6	25.0
Necrotizing enterocolitis	5	20.8
Perinatal asphyxia	4	16.6
Bronchopulmonary dysplasia	4	16.6
Congenital umbilical hernia	4	16.6
Pulmonary apoplexy	3	12.5
Osteopathia	3	12.5
Congenital heart disease	2	8.3
Posthemorrhagic hydrocephalus	1	4.2
Periventricular leukomalacia	1	4.2
Intraventricular hemorrhage	1	4.2

At 12 months postnatal age, the infants were examined using a sterile dental mirror and artificial light, prior to which they had not received food or drink for 30 min or had their teeth brushed. One trained examiner conducted all the clinical oral examinations and collected the saliva samples. For every child, an unstimulated saliva sample was collected from the dorsum of the tongue and from the labial surfaces of the upper deciduous incisors with a sterile cotton swab. Each swab was placed into a sterile tube and immediately sent to the laboratory (Protean s.r.o., Czech Republic). In the laboratory, the target genomic region containing multiple highly variable parts was amplified and labeled using polymerase chain reaction (PCR) with a mixture of universal primers. The denaturated product was then hybridized to a DNA – macroarray platform with immobilized specific probes applied in duplicates. After high – stringency washing of non – hybridized probes, the macroarrays were digitally evaluated. This test (Stoma Gene™, Protean s.r.o., Ceske Budejovice, Czech Republic) uses three independent variable genomic regions for the detection of each pathogen and therefore provides very reliable results. In this way, three cariogenic bacterial species and seven periodontal species were detected. The Stoma – Gene™ test can determine the presence of *S. mutans* (Sm), *Lactobacillus* spp. (Lsp), *Actinomyces* spp. (Asp), *Aggregatibacter actinomycetemcomitans* (Aa), *P. gingivalis* (Pg), *Tannerella forsythia* (Tf), *T. denticola* (Td), *Peptostreptococcus micros* (Pm), *Prevotella intermedia* (Pi), and *F. nucleatum* (Fn) and evaluate their quantities as follows:

1. – undetected (with < 10^3^ colony forming units (CFUs))

2. + weakly positive (with 10^3^ - 10^4^ CFUs)

3. ++ medium positive (with CFU 10^4^ - 10^5^ CFUs)

4. +++ strongly positive (with > 10^5^ CFUs).

The data were statistically analyzed using Statgraphics software distributed by Stat Point Technologies, Inc. of Warrenton, Virginia, USA. The chi-squared test and, when only a few cases were assessed in some categories, Fisher’s factorial test was used for the analyses, with p < 0.05 considered to be statistically significant. The data on the detected microbes used for the statistical comparison between the pre-term and full-term infants were reduced to just two alternatives – negative (undetected) and positive (detected, at any level). We also evaluated the levels of the detected microbes in both groups of infants. For this analysis, a scale of quantities was used: undetected, weakly positive, medium positive and strongly positive.

### Ethical considerations

Ethical approval for the investigation was obtained from the Research Ethics Committee Faculty of Medicine in Pilsen, Charles University in Prague. The study was conducted in accordance with the Helsinki Declaration of 1975, as revised in 1983. All infants were recruited from the Department of Neonatology of the Faculty Hospital in Pilsen, Czech Republic. Before the study, the legal guardians of all infants provided informed consent for their children to participate in the study.

## Results

**Figure 1:**
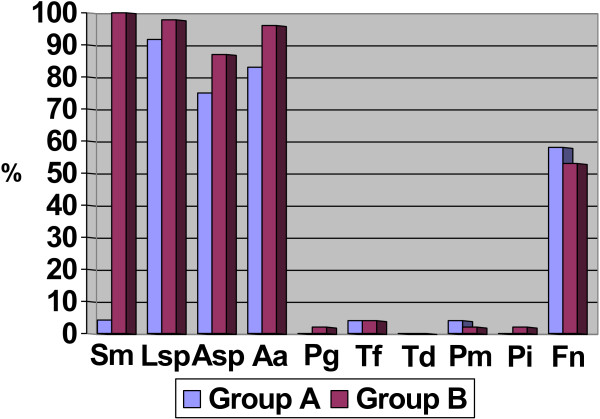
Prevalence of cariogenic and periodontal microbes.

The examined groups of infants had a high prevalence of cariogenic microbes (*S. mutans*, *Lactobacillus species* and *Actinomyces species*) and of *F. nucleatum* and *Aggregatibacter actinomycetemcomitans*. Other periodontal pathogens were detected in a small number of cases, and *T. denticola* was not detected (Figure 1). The findings concerning the particular monitoring of microbes in both groups of infants are presented in Table 3, where the absolute frequency of the individual cases as well as their percentages in the two subgroups are described.

**Table 3:** Prevalence of cariogenic and periodontal bacteria in 12-month-old infants

	**Group A**	**Group B**	**Σ**	**P - value**
**Cariogenic bacteria**				
Sm	1 (4.2%)	45 (100%)	46 (66.7%)	< 0.05
Lsp	22 (91.7%)	44 (97.8%)	66 (98.7%)	NS
Asp	18 (75%)	39 (86.7%)	57 (83%)	NS
**Periodontal bacteria**				
Aa	20 (83.3%)	43 (95.5%)	63 (91%)	NS
Pg	0 (0%)	1 (2%)	1 (1%)	NS
Tf	1 (4%)	2 (4%)	3 (4%)	NS
Td	0 (0%)	0 (0%)	0 (0%)	
Pm	1 (4%)	1 (2%)	2 (3%)	NS
Pi	0 (0%)	1 (2%)	1 (1%)	NS
Fn	14 (58.3%)	24 (53.3%)	38 (55%)	NS

### The occurrence of cariogenic microbes

One or more cariogenic microbes were detected in 91.7% of the very low birthweight infants and in all full-term infants. The most often identified microbe in the cariogenic group was the *Lactobacillus species*, which was found in 98.7% of the infants, followed by *Actinomyces species* (83% of the infants) and *S. mutans* (66.7% of the infants).

### S. mutans

In group A, only 1 of the 24 children (4.2%) was positive for *S. mutans*, while in group B, all 45 children (100%) harbored this bacteria. This difference was found to be statistically significant (chi square test-p = 0.0001).

### Lactobacillus species

Both groups of infants had high rates of *Lactobacillus species* colonization. *Lactobacillus species* were the most often detected cariogenic microbes in the group of pre-term infants. *Lactobacillus species* were detected in 22 (91.7%) of the 24 very low birthweight infants and in 44 (97.8%) of the 45 full-term infants. Fisher’s factorial test indicated a non-significant difference (p = 0.276).

### Actinomyces species

Positive values of *Actinomyces species* were detected in 18 (75%) of the pre-term infants and in 39 (86.7%) of the full-term infants. Fisher’s factorial test was used for the statistical evaluation (p = 0.318), and the results indicated that the incidence of positive values of *Actinomyces species* in the pre-term infants differs from that of the full-term infants could not be proven.

### The occurrence of periodontal pathogens

One or more periodontal pathogens were detected in 83% of the pre-term and 96% of the full-term infants. *Aggregatibacter actinomycetemcomitans* was the most commonly present periodontal pathogen found in the oral cavities of the infants enrolled in our study.

### Aggregatibacter actinomycetemcomitans

*Aggregatibacter actinomycetemcomitans* was detected in 20 (83.3%) of the pre-term infants and 43 (95.5%) of the full-term infants. The Fisher’s factorial test (p = 0.173) indicated that the differences between the groups were not significantly different.

### Fusobacterium nucleatum

*Fusobacterium nucleatum* was found in the saliva of 55% of the infants in our study. No statistically significant differences between the pre-term infants and the full-term infants were found regarding the presence of *F. nucleatum*.

*Porphyromonas gingivalis*, *Tannerella forsythia*, *Peptostreptococcus micros* and *Prevotella intermedia* were rarely found in either group of infants. The differences between the groups were not statistically significant.

### The values of the detected microbes

The weakly positive values of *Lactobacillus species* and *Actinomyces species* were the most frequent values of cariogenic microbes in the group A. *Streptococcus mutans* was strongly positive in one infant (4.2%) in group A, while it was strongly positive in 48.9% of the group B infants. In both groups, *Lactobacillus species* and *Actinomyces species* were mostly found to be weakly positive (Table 4). *Aggregatibacter actinomycetemcomitans* was found at a medium positive level in 33.3% of the group A infants and at a weakly positive level in 42.2% of the group B infants (Table 5). *Fusobacterium nucleatum* was most frequently weakly positive in both groups.

**Table 4:** Values of the cariogenic microbes

	**Sm**				**Lsp**				**Asp**			
	**Detected (d)**	**Positivity**			**Detected (d)**	**Positivity**			**Detected (d)**	**Positivity**		
**Group A (n = 24)**												
		+	++	+++		+	++	+++		+	++	+++
n_d_	1	0	0	1	22	19	3	0	18	13	4	1
% (n)	4.2	0	0	4.2	91.7	79.2	12.5	0	75.0	54.2	16.7	4.1
% (n_d_)	100.0	0.0	0.0	100.0	100.0	86.3	13.6	0.0	100.0	72.2	22.2	5.6
**Group B (n = 45)**												
		+	++	+++		+	++	+++		+	++	+++
n_d_	45	12	11	22	44	32	12	0	39	24	8	7
% (n)	100.0	26.7	24.4	48.9	97.8	71.1	26.7	0.0	86.7	53.3	17.8	15.5
% (n_d_)	100.0	26.7	24.4	48.9	100.0	100.0	72.7	27.3	100.0	61.5	20.5	17,9

**Table 5:** Values of the periodontal microbes

	**Aa**				**Fn**			
	**Detected (d)**	**Positivity**			**Detected (d)**	**Positivity**		
**Group A (n = 24)**								
		+	++	+++		+	++	+++
n_d_	20	6	8	6	14	11	2	1
% (n)	83.3	25.0	33.3	25.0	58.3	45.8	8.3	4.2
% (n_d_)	100.0	30.0	40.0	30.0	100.0	78.5	14.2	7.2
**Group B (n = 45)**								
		+	++	+++		+	++	+++
n_d_	43	19	15	9	24	18	3	3
% (n)	95.5	42.2	33.3	20.0	53.3	40.0	6.6	6.6
% (n_d_)	100.0	44.2	34.8	20.9	100.0	75.0	12.5	12.5

## Discussion

The composition of the oral microbiota varies with the age of the host. Age-related changes in the oral cavity result from tooth eruption, dietary changes, hormonal fluctuations and salivary flow. Infants are especially susceptible to microbial colonization, as specific antibodies, such a secretory immunoglobulin A, are present at relatively low levels during infancy. At birth, the oral cavity is usually void of microbes, except in cases of chorioamnionitis [11]. Rapid contamination of external and internal surfaces occurs when the fetus is exposed to the flora of the birth canal and to the external environment, especially through contact with the mother and with contaminated milk and water. The rate and extent of neonatal oral cavity colonization depends on various perinatal and neonatal factors, such as the mucosal immune system, gestational age, mode of delivery, hospitalization in an intensive care unit, type and mode of feeding and antibiotic treatment. The mucosal immune system represents the first line immune response against oral infection. Secretory IgA present in the saliva may control the oral microbes by preventing their adherence to the oral mucosa and the teeth [12]. Salivary IgA concentrations and IgA antibody specificities appear to be influenced by gestational age, which may reflect the level of the mucosal immune system [13]. Because neonates are immunologically immature, they are at risk of developing infections. Premature or low-birthweight neonates are thus at a particularly high risk of infection [14]. However, the colonization of the oral cavity after birth could be delayed by Caesarean section delivery or total parenteral nutrition [14,15].

Cariogenic microbe colonization, especially with the major pathogenic microorganism associated with dental caries (*S. mutans*), was found to occur during a window of infectivity between 19 and 31 months of age. This so-called “window of infectivity” theory of cariogenic microbe acquisition should be reevaluated using modern molecular techniques for microbial detection. Real-time PCR is a clinical tool for detecting and quantifying the presence of bacterial pathogens. Compared to traditional culturing methods, real-time PCR is fast and cost effective, but it is limited by the quality of the primers and probes chosen. These primers and probes must be sensitive enough to detect all of the target organisms yet specific enough to exclude all others [16]. Cephas et al. [17] demonstrated that 62.2% of the edentulous infants had *Streptococcus* genera in their salivary samples. Milgrom et al. [18] studied the occurrence of *S. mutans* in infants between 6 and 12 months of age and found these microbes in 48.3% of the plaque samples and in 58.3% of the saliva samples collected from their tongues. In our study, *S. mutans* was detected in all full-term one-year-old infants.

It has been speculated that birth by Caesarean section would decrease an infant’s early colonization by *S. mutans* because the immune defense system of neonates can avoid the extensive exposure to maternal microorganisms that occurs during vaginal birth [19]. Our results did not confirm speculations about the relationship of the delivery mode and the early colonization of the oral cavity with *S. mutans*. In our study, most premature infants (75%) were born via Caesarean section and still had minimal *S. mutans* colonization of the oral cavity.

The presence of *Lactobacillus species* is correlated with both active caries and an increased predisposition for future caries, as these bacteria interact with other microorganisms during their colonization. We found very high positive levels of *Lactobacillus species* (98.7%) in the saliva samples of the one-year-old infants. However, Könönen et al. [20] detected *Lactobacillus species* in only 2% of 12-month-old infants.

*Actinomyces species* play an important role in the initiation and progression of caries due to their aciduricity and ability to co-aggregate with *S. mutans*[21]. *Actinomyces species* were present in 83% of the caries-free infants in our study, which is consistent with the reported 91% prevalence of this species in one-year-old infants described by Könönen et al. [20].

Anaerobic bacterial species associated with the onset of periodontal diseases constitute an important part of the bacterial community of the mouth. The timing and type of the initial oral colonization of infants is of great importance, as it lays the groundwork for all further colonization. According to some previous studies [22,23], the colonization of the oral cavity in infants by periodontal pathogens is thought to be rare, occurring most commonly when the mother has periodontitis. Improved methods based on the PCR technique suggest that the acquisition of some microbes associated with periodontal disease occurs in the oral cavity earlier than was previously supposed. Obligate anaerobes can and do begin to colonize the oral cavities of infants prior to tooth eruption [24]. Some authors [25-27] have detected periodontal pathogens in young children, although the prevalence of these microbes has been relatively low. In our earlier study [28], periodontal pathogens (*Aggregatibacter actinomycetemcomitans, Prevotella intermedia* and *F. nucleatum*) were found in the saliva of 2.9% of newborns, 63.8% of six-month-old predentate infants and 97.8% of 12- month-old infants. Yang et al. [25] detected periodontal pathogens in 71% of children with an average age of 32.4 ± 7.5 months. In our recent study, we found that 91% of full-term one-year-old infants had periodontal pathogens. Thus, the reported prevalence of periodontal pathogens in the oral cavity varies considerably across different studies [24-27]. One of the most important periodontal pathogens is *Aggregatibacter actinomycetemcomitans*. This microbe was the most frequently detected periodontal pathogen found in our study and was present in the saliva of 83% of the very-low birthweight infants and in 96% of the full-term infants. Lamell et al. [29] detected *Aggregatibacter actinomycetemcomitans* in only 25% of infants under one year of age and, according to their work, *Aggregatibacter actinomycetemcomitans* is usually a transient colonizer of a child’s oral cavity.

*Fusobacterium nucleatum* is a quantitatively prominent component of the dental plaque and is one of the first Gram-negative species to become established in the plaque biofilm. *Fusobacterium nucleatum* is the most frequent strictly anaerobic species that exists in the oral cavity at one year of age. This microbe has been found to be present in the oral cavities of 91% of one-year-old infants and of 60% of younger infants with a mean age of 3 months [20]. Here, we found the incidence of *F. nucleatum* to be low in both groups of infants.

Cortelli et al. [26] described the initial colonization by *Prevotella intermedia* and *Tannerella forsythia* in an age group of 6 to 12 years, and *P. gingivalis* was first detected in much older individuals, aged 19 to 44 years. Cortelli et al. [27] suggested that alterations in the oral microenvironment accompanied by the eruption of teeth and the formation of the gingival sulcus provide environmental niches that are favorable for the growth of *Tannerella forsythia, Prevotella intermedia and Prophyromonas gingivalis.* Könönen et al. [20] detected these microbes, including *T. denticola* and *Peptostreptococcus micros*, in the oral cavity of 12-month-old infants, but their incidence was very low. Our findings regarding the oral colonization of these periodontal pathogens in the same age group are consistent with those of the described studies. *Treponema denticola* was not detected in the children we examined.

Oral colonization with cariogenic and periodontal pathogens in pre-term infants has not been adequately investigated. The authors [30,31] focused only on a comparison of the *S. mutans* colonization of the oral cavity in pre-term and full-term infants. Wan et al. [30] used selective tryptone-yeast-cysteine-sucrose-bacitracin agar for *S. mutans* isolation and detected *S. mutans* in 60% of full-term six-month-old infants and in over 50% of pre-term six-month-old infants. In their longitudinal study, Wan et al. [30] found that *S. mutans* colonization increased with infant age. At 12 months of age, 37% of infants harbored *S. mutans*, and there was a higher prevalence of these microbes in full-term infants than in pre-term infants, although this difference was not statistically significant. After 12 months of age, a higher prevalence of *S. mutans* was reported in the pre-term infants, but again, this difference was not statistically significant. Factors associated with *S. mutans* colonization included sweet liquids being taken to bed, frequent sugar exposure, snacking, sharing of adult food and high maternal *S. mutans* levels. In contrast, a lack of *S. mutans* colonization was connected with tooth brushing and multiple courses of antibiotics.

Seow et al. [31] studied the presence of *S. mutans* in 12-month-old infants and the differences between pre-term and full-term infants using quantitative real-time PCR techniques. They found no significant differences between these groups of infants. This may be related to the similar dietary and oral hygiene habits in both groups. The results of this research contradict the findings of our own study. In our study, we found a significant difference in the presence of oral *S. mutans* between pre-term and the full-term infants.

A number of factors could help explain the low observed occurrence of *S. mutans* in the oral cavities of the pre-term infants. Late transmission and colonization with *S. mutans* can be considered because these infants were not in frequent contact with their mothers from birth. Pre-term infants are also repeatedly treated with antibiotics, which impacts the composition of their oral microbial flora. Wan et al. [30] found that non-colonization by *S. mutans* was associated with multiple courses of antibiotics. In addition to antibiotic dosing, frequent parenteral nutrition, differences in mucosal immunity, and repeated endotracheal intubations and laryngoscopies may all affect the bacterial colonization of the oral cavities of pre-term infants. Lengthy hospitalizations of pre-term infants in intensive care units may also influence bacterial colonization. These factors may not affect the periodontal pathogens, which are much later colonizers of the oral cavity. This was confirmed in our study, as we found that the incidence of the major periodontal pathogens was similar in both study groups.

The authors are aware of certain limitations of this study. The infants’ dietary and tooth brushing histories were not obtained, a relatively small number of infants were included in the research group, and only one sample was taken from each infant for microbial detection.

## Conclusions

The data obtained in this study confirm the early transmission of cariogenic and periodontal pathogens to the oral cavities of one-year-old infants. Based on the presence of the main cariogenic microbe, *S. mutans*, significant differences were found between infants born prematurely with very low birthweights and the control group. Most of the very low birthweight infants had negative values of *S. mutans*. We found no other statistically significant differences in the presence of other microbes belonging to the cariogenic and periodontal pathogens. The presence of cariogenic microbes in the oral cavity represents a risk factor for the development of early childhood caries. Future clinical research should focus on establishing the clinical importance of periodontal pathogens in the saliva of infants.

## Abbreviations

PCR: Polymerase chain reaction; VLB: Very low birth weight; DNA: Deoxyribonucleic acid; Sm: Streptococcus mutans; Lsp: Lactobacillus spp; Asp: Actinomyces spp; Aa: Aggregatibacter actinomycetemcomitans; Pg: Porphyromonas gingivalis; Tf: Tannerella forsythia; Td: Treponema denticola; Pm: Peptostreptococcus micros; Pi: Prevotella intermedia; Fn: Fusobacterium nucleatum; CFU: Colony forming unit; IgA: Immunoglobulin A.

## Competing interests

The authors declare that there are no competing interests.

## Authors’ contributions

VM and RK contributed to the design of the study. JD performed the clinical examinations of both groups of infants. VM collected the saliva and plaque samples. VM, RK, ZB analyzed the data. All of the authors contributed to the preparation of the manuscript and approval of the final version. All authors read and approved the final manuscript.

## Pre-publication history

The pre-publication history for this paper can be accessed here:

http://www.biomedcentral.com/1472-6831/14/109/prepub
